# Bladder Neuromodulation in Acute Spinal Cord Injury via Transcutaneous Tibial Nerve Stimulation: Cystometrogram and Autonomic Nervous System Evidence From a Randomized Control Pilot Trial

**DOI:** 10.3389/fnins.2019.00119

**Published:** 2019-02-19

**Authors:** Argyrios Stampas, Kenneth Gustafson, Radha Korupolu, Christopher Smith, Liang Zhu, Sheng Li

**Affiliations:** ^1^Department of Physical Medicine and Rehabilitation, University of Texas Health Science Center at Houston, Houston, TX, United States; ^2^Department of Bioengineering, Case Western Reserve University, Cleveland, OH, United States; ^3^Department of Urology, Baylor College of Medicine, Houston, TX, United States; ^4^Biostatistics and Epidemiology Research Design Core, University of Texas Health Science Center at Houston, Houston, TX, United States

**Keywords:** spinal cord injuries, heart rate variability, neuromodulation, autonomic nervous system, neurogenic urinary bladder, transcutaneous electric stimulation

## Abstract

**Aim:** Percutaneous tibial nerve stimulation is used to decrease incontinence in chronic neurogenic bladder. We report the findings from a subset of patients in a randomized control trial of transcutaneous tibial nerve stimulation (TTNS) for bladder neuromodulation in acute spinal cord injury (SCI) in whom heart rate variability (HRV) was recorded before and after cystometrogram (CMG). The aim was to correlate autonomic nervous system (ANS) changes associated with the CMG changes after the trial using HRV analyses.

**Methods:** The study was a double-blinded sham-controlled 2-week trial with consecutive acute SCI patients admitted for inpatient rehabilitation, randomized to TTNS vs. control sham stimulation. Pre- and Post- trial CMG were performed with concurrent 5-min HRV recordings with empty bladder and during filling. Primary outcomes were changes with CMG between/within groups and associations to the HRV findings.

**Results:** There were 10 subjects in the TTNS group and 6 in the control group. Pre-trial baseline subject characteristics, blood pressures (BPs), and CMG were similar between groups. In both groups, the pre-trial systolic BP increased during filling CMG. After the trial, the control group had significantly increased detrusor pressure and counts of detrusor-sphincter dyssynergia on CMG, not seen in the TTNS group. Also, the control group did not maintain rising BP post-trial, which was observed pre-trial and remained in the TTNS group post-trial. HRV was able to detect a difference in the ANS response to bladder filling between groups. Post-trial HRV was significant for markers of overall increased parasympathetic nervous system activity during filling in the controls, not seen in the TTNS group.

**Conclusion:** Preliminary evidence suggests that TTNS in acute SCI is able to achieve bladder neuromodulation via modulation of ANS functions.

**Clinical Trial Registration:** clinicaltrials.gov, NCT02573402.

## Introduction

Neurogenic bladder develops over time after a period of spinal shock following acute spinal cord injury (SCI), leading to detrusor hyperreflexia (DH), detrusor sphincter dyssynergia (DSD), decreased bladder compliance and capacity, and increasing detrusor pressures. It is estimated that DH and/or DSD occurs in 95% of suprasacral SCI with up to 50% developing serious urologic complications (Weld and Dmochowski, 2000; Bacsu et al., 2012). The status quo for the management of neurogenic bladder in acute SCI is limited to maintaining safe pressures and volumes through a combination of timed voiding, catheterization, and anticholinergic bladder medications. Although significant reductions in morbidity and mortality have been achieved, little is done to mitigate the development of DH and DSD (Whiteneck et al., 1992). However, neuromodulation of the acute neurogenic bladder has the potential to alter the natural course of neurogenic bladder in SCI.

For example, when invasive sacral neuromodulation was performed in acute SCI, the group receiving the implanted device, under continuous operational condition, maintained normal bladder capacity, reported improved quality of life scores, and the detrusor did not develop hyperactivity (Sievert et al., 2010). The control group experienced the typical sequelae of SCI neurogenic bladder over time, including decreased bladder capacity and frequent UTIs complicated by sepsis and hospitalizations. Using similar proposed neural pathways, the afferent input from tibial nerve stimulation (TNS) improves bladder outcomes through neuromodulation of the spinal reflexes which may also prevent development of the pathologic reflexes suspected to cause DH/DSD (Gupta et al., 2015).

We hypothesized that neuromodulation of the bladder in acute SCI can be achieved with transcutaneous TNS as measured by cystometrogram and performed a double-blinded, randomized sham-controlled pilot trial of 19 subjects (Stampas et al., 2018). We reported the findings of safety and feasibility of 30-min sessions of TTNS in acute SCI as well as evidence of efficacy based on CMG findings of reduced bladder capacity and increased counts of DSD in controls compared to the TTNS group (Stampas et al., 2018). The CMG can provide information about the lower urinary tract physiology, which is only half of the story regarding the importance of an SCI neurogenic bladder treatment. The autonomic response to bladder stimulation and filling is an equally important component of this pathology that has remained elusive.

The most common precipitating stimulus of autonomic dysreflexia (AD), the only SCI cardiovascular condition to directly cause death characterized by sudden episodic increases in blood pressure (BP) in response to noxious simulation below the level of injury (Karlsson, 1999; Dolinak and Balraj, 2007) originates from the bladder (Lindan et al., 1980). In those with SCI levels of thoracic level 6 (T6) and above, AD is thought to occur in approximately 50–90% of people (Snow et al., 1978; Lindan et al., 1980; Curt et al., 1997; Sharif and Hou, 2017). Therefore, another goal of improving neurogenic bladder in SCI is to improve the response of the ANS to bladder stimuli and subsequently reduce or eliminate AD. A leading theory behind the development of AD is that the sympathetic preganglionic neurons below the level of injury undergo maladaptive neuroplasticity which leads to the reflexive release of massive amounts of norepinephrine resulting in AD (Sharif and Hou, 2017). Furthermore, pelvic afferent fibers are upregulated following SCI, of which the unmyelinated C-fibers play an essential role in eliciting AD (Hou et al., 2008). Therefore, it is imperative to understand whether neuromodulation of the bladder can impact the development of AD. To this end, heart rate variability (HRV) and BP measurements can be used to measure the ANS responses during bladder filling as well as the changes in the ANS as it relates to the developing neurogenic bladder.

Heart rate variability measurements, reflecting the ANS balance between the sympathetic (SNS) and parasympathetic nervous system (PNS), have the potential to be used as a clinical tool, requiring a non-invasive electrocardiogram (ECG) measurement using established guidelines for their recording and interpretation, producing frequency- and time-domain variables (Task Force of the European Society of Cardiology the North American Society of Pacing Electrophysiology, 1996). HRV has been found to be a reliable and reproducible measurement in SCI (Ditor et al., 2005). Recently, HRV measurements have been performed concurrently with CMG testing in chronic SCI and controls, with findings of significant differences between groups (Huang et al., 2016). The ability to detect differences in the ANS via HRV while filling the bladder during CMG may improve the understanding of the autonomic component of neurogenic bladder and the changes as it develops.

Many of the HRV analyses in SCI have focused on the frequency-domain findings, likely owing to the validation study in SCI (Ditor et al., 2005). According to the Task Force of the European Society of Cardiology and the North American Society of Pacing and Electrophysiology, the High-frequency (HF) spectral component represents cardiac parasympathetic control (Task Force of the European Society of Cardiology the North American Society of Pacing Electrophysiology, 1996). The Low-frequency (LF) spectral component has been generally accepted to represent baroreflex-mediated (sympathetic-driven) vagal outflow. The LF to HF ratio has been used to describe the cardiac sympathovagal balance. Total power in HRV reflects the range of overall variation of the sympathetic and parasympathetic nervous system. Largely under-reported in the SCI literature, the time-domain variables reflect the parasympathetic activity (NN50, pNN50, RMSSD). However, the standard deviation of all normal-to-normal intervals (SDNN) reflects the sympathetic and parasympathetic activity influencing HRV.

The aim of this exploratory study was to correlate CMG and ANS changes, specifically BPs and HRV, before and after TTNS during filling CMG in a subset of patients from the pilot randomized sham-control trial of TTNS in acute SCI (Stampas et al., 2018) who had concurrent ANS measurements. We hypothesized that TTNS could neuromodulate the bladder function and alter the response of the ANS during bladder filling with routine use. Specifically, we hypothesized that (1) TTNS would prevent the harmful CMG changes that would be found in the control group and (2) that ANS response to bladder filling would differ between groups based on HRV and BP recordings.

## Materials and Methods

Patients with acute SCI were recruited for this study from July 2016 to October 2017 during admission to inpatient rehabilitation. Patients were screened using the electronic medical record (EMR) and then approached to complete verification of the inclusion/exclusion criteria and consent. Inclusion criteria were: (1) acute traumatic SCI; (2) 18–65 years old; (3) within 6 weeks of injury; (4) neurologic level of injury T9 and rostral, to avoid damage to the lower motor neurons (LMNs) of the bladder. Exclusion criteria were: (1) prior central nervous system disorder; (2) peripheral neuropathy; (3) premorbid genitourinary diagnoses; (4) known LMN injury to the bladder or its potential due to multitrauma; (5) ventilator assistance for respiration due to the challenges in performing the cystometrogram in these subjects. See Table 1 for subject details.

**Table 1:** Baseline demographics.

	Control (*n* = 6)	TTNS (*n* = 10)	*p*-value
	**Mean (SD)**		
Age (years)	51.8 (9.6)	38.1 (14.2)	0.06
Duration of injury (days)	19.5 (6.7)	22.7 (9.1)	0.45
Admission FIM motor	15.5 (5.5)	17.5 (5.3)	0.45
Admission FIM bladder	1 (0)	1 (0)	
Admission FIM cognition	30.3 (4.3)	27.3 (6.1)	0.41

	**Frequency (%)**		

Male	1 (17%)	7 (70%)	0.12
Tetraplegia	4 (67%)	4 (40%)	0.61
Neurologic level			0.47
C1-4	3 (50%)	4 (40%)	
C5-8	1 (17%)	0	
T1-T4	1 (17%)	5 (40%)	
T5-T9	1 (17%)	1 (10%)	
AIS severity			0.46
A	3 (50%)	8 (70%)	
B	0	1 (10%)	
C	2 (33%)	1 (10%)	
D	1 (17%)	0	

*TTNS, transcutaneous tibial nerve stimulation group; FIM, functional independence measure; AIS, American Spinal Injury Association (ASIA) Impairment Scale; AIS A, complete injury; AIS B, motor complete, sensory incomplete injury; AIS C, motor incomplete injury with less than half of the muscles below the level of injury with antigravity strength; AIS D, motor incomplete with at least half the muscles below the level of injury with antigravity strength.*

### Study Design

**FIGURE 1:**
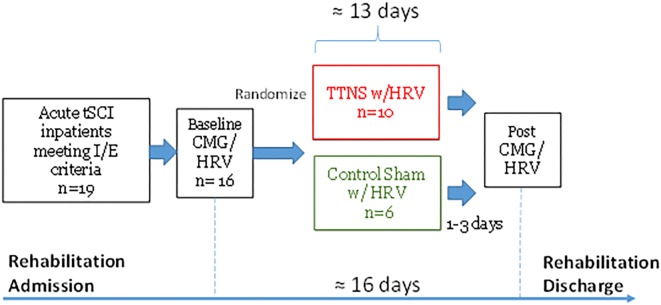
Study Timeline. tSCI, traumatic spinal cord injury; I/E, inclusion/exclusion criteria; CMG, cystometrogram; HRV, heart rate variability; TTNS, transcutaneous tibial nerve stimulation.

Upon consent, baseline CMG was performed and subjects were randomized into TTNS and control groups in a 2:1 ratio, stratified by areflexic bladder to ensure a balanced distribution between the two groups (Figure 1). After this pre-trial CMG, TTNS versus control stimulation was performed and upon completion, post-trial CMG was performed within 3 days after the final intervention. Following the CONSORT 2010 guidelines, randomization and experiments were performed by the research assistant, while the principle investigator and subjects were blinded to treatment allocation. Further details can be found in the complete pilot trial publication (Stampas et al., 2018). This study was approved by the Institutional Review Board and is registered with clinicicaltrials.gov: NCT02573402.

### The Interventions

Those in the TTNS group received 30 min of TTNS per day for 10 days within a 16-day period. TTNS was applied to the right leg with the negative electrode behind the internal malleolus and the positive electrode 10 cm. superior to the negative electrode, verified by big toe flexion with rising current intensity (Amarenco et al., 2003). Stimulation frequency of 10 Hz and 200 μs duration was used with current intensity increased until toe flexion, then lowered (sub-motor) for 30 min at constant stimulation (Amarenco et al., 2003; Finazzi Agrò et al., 2005). Those in the control group received sham stimulation in which the electrodes were placed and the stimulator was activated until toe flexion, but then immediately reduced to zero intensity. The display on the device was covered in both arms to prevent subjects from reading the current intensity.

### The Assessment

The urodynamic methodology complied with International Continence Society recommendations (Schafer et al., 2002). Subjects were brought to the urology procedure suite, transferred to the examination table, and catheterized to empty the bladder. Disposable adhesive electrodes were attached to each electrocardiogram (ECG) lead and the white “right arm” and black “left arm” electrode leads were placed along the 1st intercostal spaces in the subclavicular area on the right and left chest wall, respectively. Subjects were asked to remain motionless without speaking as the 5-min ECG recordings were collected using the HRV heart rhythm scanner (Biocom 5000 Wireless ECG Recorder, Biocom Technologies, Poulsbo, WA, United States). After the CMG catheter was inserted and waiting 5 min with emptied bladder, ECG recordings started with Empty bladder recordings. At the initiation of bladder filling, Filling 1 recordings began for 5 min, followed by Filling 2 recordings for 5 min. ECG signals were saved for off-line HRV analyses.

Cystometry was performed with the patient supine through a double lumen 7 Fr. catheter with computerized analysis of the results using normal saline at 25 to 30°C with a filling rate of 40 ml per minute, terminating at 600 ml of bladder capacity or sooner as clinically indicated. Clinical indications for early termination of the CMG includes overflow leaking and AD. Filling 1 was 0–200 cc followed by Filling 2 with approximately 200–450 cc of infused saline, based on the volumes infused during the 5-min HRV recordings. Filling phases were used because the 5-min HRV recording could not record the entire CMG. The same procedures were followed for both pre- and post- TTNS trial measurements, with bladder empty and bladder filling, at a similar time of day. Blood pressures were measured with Welch Allyn^®^ Vital Signs Monitor 300 before and during filling, every 5 min or sooner as clinically indicated. A rise of systolic BP greater than or equal to 20 mmHg was defined as AD (Curt et al., 1997). The Numerical Pain Scale (NPS) was recorded for each patient at the time of CMG as a possible HRV confounding variable (Karri et al., 2017).

### Data Analyses

From the larger pilot trial (Stampas et al., 2018) the primary outcomes were safety and feasibility measures and the secondary outcomes were the within- and between-group CMG changes after TTNS. Measurements included: maximum detrusor pressure (cmH_2_O); bladder capacity [maximum volume infused (ml)]; frequency of DSD and DH (count); volume at first involuntary detrusor contraction (ml); and subjective measures of first sensation and desire to void [based on infused volume of saline (ml)]. DH was defined as a non-volitional increase in detrusor pressure of at least 6 cm H_2_O (Kaplan et al., 1991). DSD is defined as the presence of involuntary contractions of the external sphincter during detrusor contractions (Blaivas et al., 1981). Factors that may affect the bladder including medication use in the 24 h prior to the CMG and presence of an indwelling catheter were recorded and used in the analyses.

For this study cohort with ANS measures from the larger pilot trial, the primary outcome measures were the ANS differences with bladder filling within and between groups based on HRV and BP changes. Kubios HRV analysis software (University of Eastern Finland, Joensuu, Finland) was used offline to analyze the ECG recordings via time and frequency domains. Time-domain parameters included Mean R–R interval (RR is the interval between successive Rs on the QRS complex of the ECG wave), Mean HR (heart rate), STDHR (standard deviation of HR), SDNN (standard deviation of normal to normal R–R intervals), RMSSD (root mean squared of successive differences), NN50 (pairs of successive R–R beat lengths that differ by more than 50 ms), and pNN50 (the proportion of NN50 for total number of beats). Frequency domain measurements included low-frequency (LF), high-frequency (HF), the ratio LF/HF, and total power (the sum of power of all frequency bands, reflecting the range of variation of the overall autonomic regulation).

### Statistical Methodology

**FIGURE 2:**
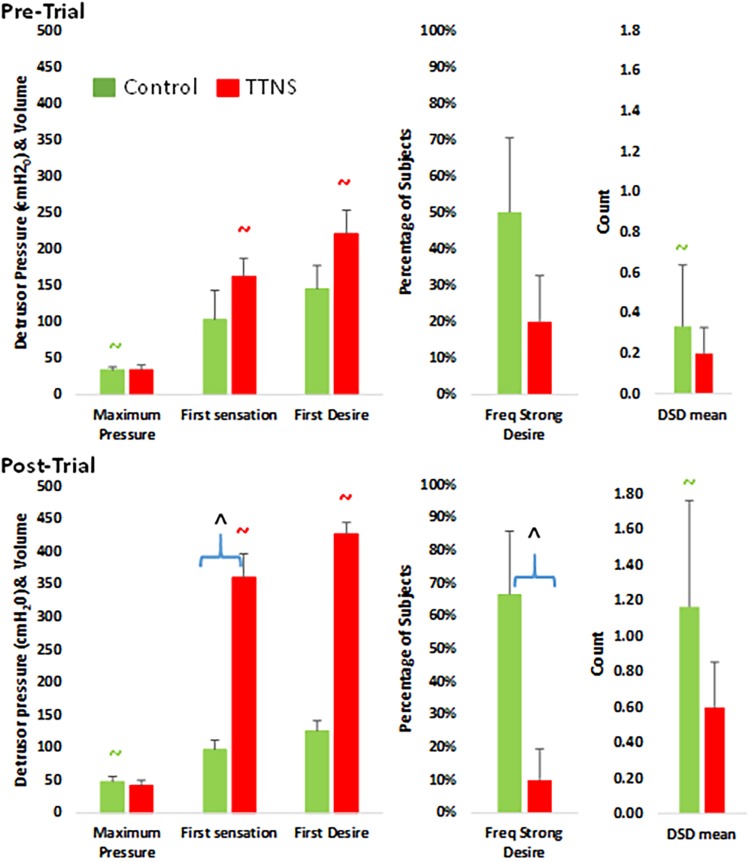
Bar graphs of significant differences between control (green) and TTNS (red) groups, pre- **(top)** and post-trial **(bottom)**. DSD, detrusor-sphincter dyssynergia. All symbols are significant *p* < 0.05 and color-coded: ^∧^between group difference; ∼ pre- vs. post-difference.

**FIGURE 3:**
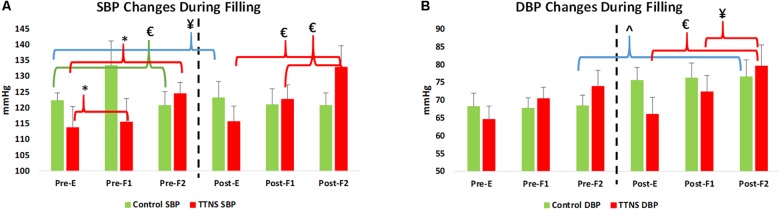
Significant blood pressure (BP) changes during bladder filling, pre- and post-trial. Dashed line represents transition to post-trial testing, red brackets and green brackets are within group differences in the TTNS and controls, respectively. Blue brackets are pre- post-trial differences. **(A)** In both groups, SBP increases with filling pre-test. Post-test, this is only seen in the TTNS group. **(B)** Diastolic BP is significantly different in the controls post-trail compared to pre-trial. Pre, pre-trial; Post, post-trial; E, empty bladder; F1, filling phase 1; F2, filling phase 2; TTNS, transcutaneous tibial nerve stimulation; SBP, systolic blood pressure; DBP, diastolic blood pressure; ^¥^*p* = 0.04; ^∗^*p* = 0.01; £*p* < 0.01; ˆ*p* = 0.03.

**FIGURE 4:**
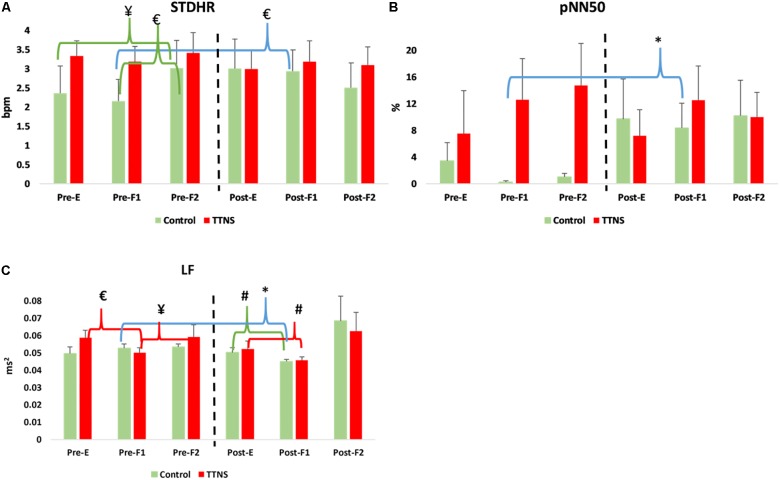
Significant HRV changes during bladder filling, pre- and post-trial. Dashed line represents transition to post-trial testing, red brackets and green brackets are within group differences in the TTNS and controls, respectively. Blue brackets are pre- post-trial differences. **(A)** STDHR increases pre-stimulation in the controls, not seen post-test. **(B)** pNN50 increases in the controls post-test during the first filling phase. **(C)** Decrease in LF during initial filling is maintained in the TTNS group post-trial. Pre, pre-trial; Post, post-trial; E, E = empty bladder; F1, filling phase 1; F2, filling phase 2; TTNS, transcutaneous tibial nerve stimulation; STDHR, standard deviation of heart rate; bpm, beats per minute; pNN50, the proportion of pairs of successive R–R beat lengths that differ by more than 50 ms (NN50) for total number of beats. LF, low frequency; ms^2^, milliseconds squared; ^¥^*p* = 0.04; ^#^*p* = 0.03; ^∗^*p* = 0.01; £*p* < 0.01.

Descriptive statistics were provided for baseline demographics for the control and TTNS groups, with comparisons using Wilcoxon Rank Sum and Fischer’s exact test for continuous and categorical data, respectively (Table 1). We compared the CMG outcomes before and after the trial within and between the two groups (Figure 2 and Table 2). For between group comparison, Wilcoxon rank sum and Fisher’s exact tests were performed on continuous and categorical variables, respectively. For within group comparison before and after the trial, multilevel mixed-effects linear regression was performed controlling for subject and duration of injury variability for continuous variables, and multilevel mixed-effects logistic regression was performed for categorical variables. Using similar statistical methods used in the CMG analyses, measures of the ANS at the time of CMG after the bladder was emptied (Empty Bladder) were performed between groups and within groups pre-/post-trial (Table 3). Between group ANS difference during Filling 1 (first 5 min of CMG filling to approximately 200 cc) and Filling 2 (next 5 min of CMG filling to approximately 450 cc) were performed using Wilcoxon rank sum test (Table 4). Multilevel mixed-effects linear regression was used to compare ANS differences within the groups pre- and post-trial, as well as the ANS changes from empty to Filling 1 to Filling 2 pre- and post-trial (Figure 3, 4). Finally, CMG variables that changed significantly were added as the dependent variables in multilevel mixed-effects linear modeling with the ANS variables as the independent variables. HRV variables that had significant pre-trial differences between groups were excluded. As an exploratory study, no adjustments were made for multiple comparisons, with plans to confirm all results with a larger scale study. Stata 14.0 (StataCorp., 2015) was used for the analyses, with *p* < 0.05 set for significance.

**Table 2:** Cystometrogram data.

	Pre-trial			Post-trial			Pre vs. post	
	Control (*n* = 6)	TTNS (*n* = 10)		Control (*n* = 6)	TTNS (*n* = 10)		Control (*n* = 6)	TTNS (*n* = 9)
**Variables**	**Mean (SD)**		***p-*value**	**Mean (SD)**		***p*-value**	***p*-value**	
Maximum detrusor pressure (cmH_2_O)	33 (12)	34 (21)	0.91	48 (21)	42 (27)	0.55	**0.04**	0.11
Bladder capacity (ml)	566 (88)	589 (31)	0.79	465 (171)	548 (115)	0.23	0.06	0.1
Freq. detrusor Hyperactivity	3.5 (3.8)	1 (1.5)	0.16	1.2 (1.6)	1.1 (1.4)	0.95	0.17	0.47
Freq. of detrusor-sphincter dyssynergia	0.33 (0.82)	0.2 (0.42)	1	1.2 (1.6)	0.6 (0.8)	0.55	**0.01**	0.23
	***N* (%)**			***N* (%)**				
Presence of:								
Involuntary contraction	4 (67%)	5 (50%)	0.63	4 (67%)	6 (60%)	1	0.92	0.63
First sensation	4 (67%)	4 (40%)	0.61	4 (67%)	4 (40%)	0.61	0.34	0.28
First desire	4 (67%)	3 (30%)	0.3	4 (67%)	2 (20%)	0.12	0.4	0.07
Strong desire	3 (50%)	2 (20%)	0.3	4 (67%)	1 (10%)	**0.04**	0.77	0.09
Detrusor areflexia	2 (33%)	5 (50%)	0.63	2	4	1	0.92	0.63
Spinal shock	1 (17%)	2 (30%)	1	0	1	1	0.32	0.32
Autonomic dysreflexia	0	3 (30%)	0.25	0	1 (11%)	1	NA	0.3
Indwelling catheter	2 (33%)	4 (40%)	1	3	4	1	0.42	0.15
Bladder medication	0	0		0	0		-	-
Spasm medication	4 (67%)	2 (20%)	0.12	3	3	0.61	0.96	0.97
	**Subgroup analysis of those with sensation/contraction Mean volumes in ml (SD)**							

First involuntary contraction	226 (168)	304 (81)	0.46	258 (258)	258 (105)	0.52	0.06	0.96
First sensation	103 (116)	162 (89)	0.39	97 (41)	361 (131)	**0.02**	0.96	**0.01**
First desire	145 (91)	221 (127)	0.29	126 (43)	427 (78)	0.06	0.6	**0.03**
Strong desire	262 (262)	341 (28)	0.56	285 (179)	482	0.48	0.265	**-**

*SD Tables 2–4.”, standard deviation; Freq., frequency; TTNS, transcutaneous tibial nerve stimulation; cmH_2_O, centimeters pressure of water; ml, milliliters; DH, detrusor hyperreflexia; DSD, detrusor-sphincter dyssynergia; AD, autonomic dysreflexia. Significant difference of bold values *p* < 0.05.*

**Table 3:** Autonomic nervous system data, empty bladder significant differences.

	Pre-trial			Post-trial			Pre vs. post	
	Control (*n* = 6)	TTNS (*n* = 10)		Control (*n* = 6)	TTNS (*n* = 9)		Control (*n* = 6)	TTNS (*n* = 9)
**Variables**	**Mean (SD)**		***p*-value**	**Mean (SD)**		***p*-value**	***p*-value**	
Systolic BP	111 (8)	110 (17)	0.95	123 (14)	116 (15)	0.44	**0.04**	0.28
SDNN (ms)	23 (16)	45 (20)	**0.02**	38 (32)	38 (22)	0.81	**0.02**	**0.04**
Total power (ms^2^)	456 (509)	2300 (2366)	**0.02**	1838 (2495)	1524 (1579)	0.81	**0.03**	**0.03**

*ms, milliseconds, bpm, beats per minute; BP, blood pressure; SDNN, standard deviation of normal to normal R–R intervals. Significant difference of bold values p < 0.05.*

**Table 4:** Autonomic nervous system, filling cystometrogram data comparisons, significant differences.

	Pre-trial						Post-trial						Pre vs. post			
	Control (*n* = 6)		TTNS (*n* = 10)		Between group difference		Control (*n* = 6)		TTNS (*n* = 9)		Between group difference		Control (*n* = 6)		TTNS (*n* = 9)	
	Mean/SD				*p*-value		Mean/SD				*p*-value		*p*-value			
	F1	F2	F1	F2	F1	F2	F1	F2	F1	F2	F1	F2	F1	F2	F1	F2
DBP (mmHg)	68/8	68/7	7/9	74/12	0.7	0.6	76/10	77/11	72/13	80/16	0.3	0.8	0.2^*^	**0**.**03**	0.5	0.2
SDNN (ms)	20/11	30/16	43/21	49/31	**0**.**02**	0.2	32/16	29/20	37/17	40/21	0.8	0.3	**<0**.**001**	0.8	0.5	0.4
STDHR (bpm)	2/2	3/2	3/1	3/2	0.07	0.3	3/1	3/2	3/2	3/2	0.4	0.5	**<0**.**001**	0.3	0.8	0.3
RMSSD (ms)	11/5	15/7	31/29	36/35	**0**.**04**	0.1	31/28	30/31	29/19	27/16	0.8	0.8	**0**.**02**	0.1	0.8	0.4
NN50 (count)	1/2	4/6	28/57	31/55	0.1	0.1	20/25	25/42	20/26	21/33	1	1	**0**.**02**	0.1	0.5	0.4
pNN50 (%)	0.3/0.5	1/2	13/21	15/21	0.1	0.1	8/10	10/14	13/16	10/12	0.8	1	**0**.**01**	0.05	1	0.4
LF (ms^2^)	0.05/0.006	0.1/0.004	0.05/0.01	0.06/0.02	0.2	0.4	0.04/0.003	0.07/0.04	0.05/0.01	0.06/0.03	0.7	0.9	**0**.**01**	0.2	0.3	0.8
Total power (ms^2^)	476/580	712/512	1582/1268	2222/3308	**0**.**02**	0.2	853/672	599/644	1508/1298	1824/1888	0.5	0.2	**0**.**04**	0.6	1	0.7

*F1, filling period 1; F2, filling period 2; ms, milliseconds; bpm, beats per minute; SDNN, standard deviation of normal to normal R–R intervals; STDHR, standard deviation of heart rate; RMSSD, root mean squared of successive differences; NN50, pairs of successive R–R beat lengths that differ by more than 50 ms; pNN50, the proportion of NN50 for total number of beats; LF, low frequency; ^∗^Wilcoxon rank-sum test used. Significant difference of bold values p <0.05.*

## Results

### Pre-trial

There were six control and 10 TTNS subjects that had HRV analyses performed from within the TTNS trial. Due to technical difficulties, we were unable to perform HRV on one subject in the TTNS group post-trial. There were no baseline demographic differences between groups in terms of age, duration of injury, sex, functional independence measure (FIM) scores on admission, levels of injury, and SCI severity (Table 1).

There were also no Pre-Trial differences on CMG between groups regarding maximum detrusor pressure, bladder capacity, frequency of DH and DSD, the occurrence of AD and filling sensations, and the volume of saline infused at the time of these sensations (Figure 2 and Table 2).

Although there were no group differences in BPs during Pre-Trial measurements with Empty Bladder (Figure 3 and Table 3), there was increased overall ANS activity in the TTNS group compared to the control group as reflected by SDNN and total power (*p* = 0.02 for both) (Table 3). This was also observed during filling CMG at Filling 1, with increased SDNN (*p* = 0.02) and total power (*p* = 0.02) in the TTNS group compared to controls (Table 4). The TTNS group also had increased RMSSD (*p* = 0.04) reflecting parasympathetic activity during Filling 1 compared to the controls (Table 4).

Both groups increased SBP during bladder filling on Pre-Trial CMG (Figure 3A). In the control group during Pre-trial CMG from Filling 1 to Filling 2, there was increased parasympathetic activity (increased mean RR, decreased mean HR, *p* = 0.006 for both; increased STDHR, *p* = 0.008) and increased overall ANS activity (SDNN, *p* = 0.005) (Table 4). STDHR also significantly increased from Empty Bladder to Filling 2, *p* = 0.035 (Figure 4A). In the TTNS group, mean RR decreased from Filling 1 to Filling 2 and from Empty Bladder to Filling 2, *p* = 0.024, 0.006, respectively. Sympathetic tone decreased in the TTNS group as reflected by LF from Empty Bladder to Filling 1 (*p* = 0.004) and increased from Filling 1 to Filling 2 (*p* = 0.04, Figure 4C), and parasympathetic tone decreased (HF, *p* = 0.045) from Empty Bladder to Filling 2.

### Post-trial

After the trial, the control group significantly increased maximum detrusor pressure (mean, SD), from 33 ± 12 to 48 ± 21 cmH_2_O (*p* = 0.04), and increased DSD events from 0.33 ± 0.82 to 1.2 ± 1.6 (*p* = 0.01) (Figure 2 and Table 2). There were also more in the control group (*n* = 4) with strong desire to void compared to the TTNS group (*n* = 1, *p* = 0.04). Comparing pre- and post-trial, there were no significant changes in the presence of involuntary bladder contractions and filling sensations in both groups. However, in those with filling sensation, the first sensation of filling was higher in the TTNS group (361 ± 131 ml) compared to the control group (97 ± 41 ml, *p* = 0.02). Furthermore, the TTNS group significantly increased the volumes needed for first sensation, from 162 ± 89 to 361 ± 131 ml (*p* = 0.01), as well as first desire to void, from 221 ± 127 ml to 427 ± 78 ml (*p* = 0.03).

After the trial, there were several ANS differences. In the control group, there was a significant rise in the resting SBP with empty bladder, from 111 ± 8 to 123 ± 14 mmHg (*p* = 0.04, Figure 3A and Table 3). The control group also significantly increased DBP during Filling 2, from 68 ± 7 to 77 ± 11 mmHg (*p* = 0.03, Figure 3A and Table 4). Overall ANS variability increased in the control group before and after the trial as reflected by the changes in SDNN and total power, *p* = 0.02 and 0.03, respectively. The control group was observed to have an overall increase in ANS activity (SDNN, total power) and PNS activity (STDHR, RMSSD, NN50, pNN50, Figure 4B) and a decrease in sympathetic tone (LF, Figure 4C) during Filling 1 (Table 4).

In the TTNS group, post-trial CMG was significant for increasing SBP during filling, from Filling 1 to Filling 2 (*p* = 0.003), as well as from Empty Bladder to Filling 2 (*p* < 0.001, Figure 3A). DBP also increased from Filling 1 to Filling 2 (*p* = 0.042) and from Empty Bladder to Filling 2 (*p* < 0.001, Figure 3B). This was not seen in the control group after the trial. The control groups demonstrated increased parasympathetic activity from Filling 1 to Filling 2 (Mean RR, *p* = 0.02; Mean HR, *p* = 0.012; HF, *p* = 0.017). In the TTNS group, there was decreased sympathetic activity from Empty Bladder to Filling 1 (LF, *p* = 0.032, Figure 4C).

### Significant CMG Variables Associated to ANS Variables

In the controls, increasing parasympathetic activity was associated to: (1) increasing maximum detrusor pressure (as NN50 increased, maximum detrusor pressure increased by 0.23 cmH_2_O, *p* = 0.02, 95%CI 0.04–0.43; as pNN50 increased, maximum detrusor pressure increased by 0.78 cmH_2_O, *p* = 0.026, 95% CI 0.09–1.46); (2) volume to first sensation and first desire increased with increasing HF (as HF increased, volume to first sensation increased (*p* = 0.021, 95%CI 32 387) and volume to first desire increased (*p* = 0.03, 95%CI 23 397).

In the TTNS group, as LF/HF increased, DSD decreased by 0.17 (*p* = 0.009, 95%CI -0.3 -0.04). As HF increased by 0.1, volume of first sensation decreased by 158 ml (*p* = 0.021, 95%CI -292 -24). Increasing volume to first desire was significantly associated to several ANS variables: (1) as DBP increased, volume increased by 6.97 ml (*p* < 0.001, 95%CI 6.95–6.99); as SDNN increased, volume increased by 7.2 ml (*p* = 0.001, 95%CI 2.9–11.6); as NN50 increased, volume increased by 5.25 ml (*p* < 0.001, 95%CI 5.25–5.25); as total power increased, volume increased by 0.035 (*p* < 0.001, 95%CI 0.035–0.035). Volume to first desire decreased with increasing HF (*p* < 0.001, 95%CI -249 -127) and increasing LF (*p* < 0.001, 95%CI -203 -201).

## Discussion

In this cohort of subjects from the larger pilot trial, we observed that the previous findings of improved CMG parameters in the TTNS group were associated with paralleled ANS changes. Our concomitant findings from BP and 5-min ECG recordings with HRV analysis add to the paucity of knowledge in the developing SCI neurogenic bladder and provide insight to the effects of bladder neuromodulation with TTNS on the autonomic nervous (ANS) system response to bladder filling.

The postulated mechanism of action of percutaneous TNS and sacroneuromodulation is that stimulation of the peripheral sensory afferent fibers block competing abnormal visceral afferent signals from the bladder and prevent the reflexive, efferent motor response resulting in detrusor hyperactivity and dysynergia (Sanford and Suskind, 2016). We postulated that this mechanism can be achieved with TTNS, impacting overall sensation from the bladder, leading to prolonged times to sensation and decreased overall reflexive efferent responses causing DSD and potentially DH. The findings from our pilot data support this mechanism, with TTNS group findings of delayed bladder filling sensations and subsequent decreased reflexive motor efferent activity represented by fewer events of DSD during bladder filling. With the addition of the ANS measurements, we sought to describe the ANS response to bladder filling with TTNS compared to controls.

Similar to the reported increase in systolic BP in non-SCI subjects and chronic tetraplegia approaching maximal bladder capacities, we found an increase in SBP as the bladder fills in both the controls and TTNS group Pre-trial (Figure 3A) (Huang et al., 2016). Over time, this physiologic response is lost in the control group, but is maintained in the TTNS group (Figure 3A). Importantly, there were no pre- post-trial differences in ANS measures in the TTNS group (Figure 4). This may suggest that the neuromodulation from TTNS not only maintains bladder mechanical responses, but also the sensory afferent responses which are communicated to the ANS.

Unlike the TTNS group after the trial, ANS measures were able to detect several changes in the control group. The control group displayed evidence of increased parasympathetic activity during early filling (NN50, pNN50) and decreased sympathetic activity (LF) with subsequent decreased HR and increased RR during late filling. This may explain the lack of increased SBP during filling which was seen pre-trial (Figure 3A). Furthermore, these findings suggest that the neurogenic bladder sensory afferents may trigger an increased parasympathetic response before the normal capacity to void is achieved.

In the control group, mixed-effects linear modeling was significant for increasing volume to first sensation and first desire to void associated to increasing parasympathetic activity measured by HF. The opposite association was observed in the TTNS group, with increasing HF associated to reduced volumes to first sensation and first desire to void. Although the meaning of this relationship remains to be determined, that opposite findings were observed in the control and intervention group are noteworthy suggesting that HRV differences are associated with CMG differences and that TTNS affects the ANS response to bladder filling. Further research with larger sample sizes is warranted to clarify these relationships.

This study also highlights the need to perform urodynamic evaluations in all people with SCI. We found that completeness of injury (AIS A) did not preclude sensation during bladder filling or the desire to void during CMG. This study, as well as the findings from others, demonstrate that physical exam, including the International Standards for Neurological Classification of SCI (ISNCSCI) exam, poorly correlate with CMG findings of lower urinary tract dysfunction, even during the acute phase of injury (Weld and Dmochowski, 2000; Patki et al., 2006; Bellucci et al., 2013). Furthermore, there are very few ways to measure changes in the ANS function and response in the human neurogenic bladder, and those that exist are limited to specialized laboratory tests, including measurements of neurotransmitters and other messaging proteins (Ochodnicky et al., 2013). We propose that the addition of feasible, clinic-setting measurements of BP and HRV to measure ANS activity during serial CMG can increase the knowledge of the developing neurogenic bladder and the impact of neuromodulation. While CMG provides us with reflexive and mechanical responses, BP and HRV data provides ANS-mediated neurophysiologic responses to bladder filling.

There are several limitations to this study. The study was powered primarily based on sample size of convenience to measure safety and feasibility of TTNS in acute SCI rehabilitation, not CMG and HRV parameter changes. Findings from these pilot trials will help develop larger trials. Although baseline BP recordings were similar between groups, randomization did not balance the overall ANS activity as measured by SDNN and total power, significantly higher in the TTNS group at baseline. Also, HRV measurements may be influenced by time of day and diet, perhaps requiring a more controlled environment to precisely measure differences. Furthermore, neurologic and physiologic changes are expected to be seen in acute SCI. Serial CMGs and HRV measurements have not been performed in acute SCI and given the low numbers in the pilot trial, it is unclear how representative the changing values before and after the trial are due to TTNS versus natural history of acute SCI. That a significant difference exists between the two groups supports the hypothesis that TTNS can modulate the ANS and warrants further research. Long-term sustainability of efficacy can only be speculated based on this 2-week protocol with outcomes measured within 3 days of trial completion. Finally, the use of HRV in neurogenic bladder is novel and the analyses conducted are likely an over-simplification of the PNS and SNS response to bladder filling. The HRV variables measure different aspects of the ANS variability and therefore have differing responses. Much like the research in orthostatic hypotension in the non-SCI population, a composite of HRV variables utilizing mathematical modeling may be necessary to define correlates to CMG findings (Sannino et al., 2015). Importantly, HRV was able to detect a difference between groups. Further research is necessary to better understand the utility and knowledge gained from HRV during CMG.

## Conclusion

Preliminary evidence suggests that TTNS in acute SCI is able to achieve bladder neuromodulation based on stable CMG findings and lack of morbid changes in the TTNS group compared to controls. The morbid CMG changes in the control group were associated with HRV markers of increased parasympathetic tone and corresponding BP findings. Further research is needed to understand the neuromodulation effects of TTNS in acute SCI neurogenic bladder and the response of the ANS during bladder filling as measured by BP and 5-min ECG recordings with HRV analyses.

## Ethics Statement

This study was carried out in accordance with the recommendations of University of Texas Health Science Center at Houston and TIRR Memorial Hermann with written informed consent from all subjects. All subjects gave written informed consent in accordance with the Declaration of Helsinki. The protocol was approved by the UTHealth IRB committee.

## Author Contributions

AS contributed to the conception, the study design, the acquisition, the analysis, the interpretation of data, and the preparation of the manuscript. KG contributed to the study design and the editing of the manuscript. RK contributed to the acquisition of data and the editing of the manuscript. CS contributed to the study design, the interpretation of data, and editing of the manuscript. LZ contributed to the study design, the analysis, the interpretation of data, and the statistical writing. SL contributed to the interpretation of data and the editing of the manuscript.

## Conflict of Interest Statement

The authors declare that the research was conducted in the absence of any commercial or financial relationships that could be construed as a potential conflict of interest.
